# Quantitative Determination of Fibrinogen of Patients with Coronary Heart Diseases through Piezoelectric Agglutination Sensor

**DOI:** 10.3390/s100302017

**Published:** 2010-03-16

**Authors:** Qinghai Chen, Xing Hua, Weiling Fu, Dongbo Liu, Ming Chen, Guoru Cai

**Affiliations:** 1 Laboratory, The Clinical Experimental Base of Biosensor and Microarray, The Center of Molecule and Gene Diagnosis, Southwest Hospital, Third Military Medical University, Chongqing 400038, China; E-Mails: huaxing525@yahoo.com.cn (X.H); Liudong_bo@tom.com (D.L.); chengming1971@yahoo.com.cn (M.C.); 2 The 26th Electronics Research Institute, National Information Industry Department, Chongqing 400060, China; E-Mail: guorucai1954@sina.com (G.C.)

**Keywords:** piezoelectric sensor, fibrinogen in plasma, coronary heart diseases, agglutination reaction

## Abstract

Fibrinogen can transform fibrin through an agglutination reaction, finally forming fibrin polymer with grid structure. The density and viscosity of the reaction system changes drastically during the course of agglutination. In this research, we apply an independently-developed piezoelectric agglutination sensor to detect the fibrinogen agglutination reaction in patients with coronary heart diseases. The terminal judgment method of determining plasma agglutination reaction through piezoelectric agglutination sensor was established. In addition, the standard curve between plasma agglutination time and fibrinogen concentration was established to determinate fibrinogen content quantitatively. The results indicate the close correlation between the STAGO paramagnetic particle method and the method of piezoelectric agglutination sensor for the detection of Fibrinogen. The correlation coefficient was 0.91 (*γ* = 0.91). The determination can be completed within 10 minutes. The fibrinogen concentration in the coronary heart disease group was significantly higher than that of the healthy control group (P < 0.05). The results reveal that high fibrinogen concentration is closely correlated to the incurrence, development and prognosis of coronary heart diseases. Compared with other traditional methods, the method of piezoelectric agglutination sensor has some merits such as operation convenience, small size, low cost, quick detecting, good precision and the common reacting agents with paramagnetic particle method.

## 1. Introduction

Fibrinogen (Fib), a blood coagulating protein with its highest content in plasma, is one of the acute phase proteins, synthesized by liver cells and megakaryocytes. Fib is closely associated with consumption coagulopathy, liver diseases, nephropathy syndrome, cardiovascular diseases, diabetes and malignant tumors, *etc*. High-levels of Fib is one of the dangerous factors contributing to coronary heart diseases [1–6]. Previous reports often focused on hemorrhagic diseases, thromboembolic diseases and recurrent spontaneous abortions caused by genetic variation of Fib [7–8]. More attention needs to be paid to Fib determination in the clinical field.

In principle, there are three types of Fib determination methods—physical-chemical methods (thermal/salt precipitation method, *etc*.), congealable protein methods and immunology methods [9–14]. More recently, with the popularity of automatic coagulometers, the application of paramagnetic particle methods has gradually increased. Although the thermal/salt precipitation method possesses the largest application scope, specificity is not ideal because the results are easily influenced by other proteins in the specimen. The congealable protein method reflects the blood agglutination function of Fib, but the method is comparatively complex and time-wasting. Therefore, this method is only applicable to establish reference scheme. The immunology methods, including the immune turbidimetry method, single diffusion method and ELISA, adopt polyclonal antibodies against Fib to react. However, because of common antigen determinants among fibrin monomer, fibrin degradation product (FDPS) and abnormal Fib, the specificity is not ideal. The results using an automatic instrument, especially for the paramagnetic particle method, is precise, rapid and well-reproducible, but the instrument is expensive and the determination cost is high [15].

Piezoelectric quartz sensor technology is a new kind of sensor detection technology. The oscillation frequency of quartz crystal is highly sensitive to changes of mass surface on the crystal and changes of physical properties of the reaction system, such as density, viscosity and electrical conductivity. The piezoelectric quartz sensor possesses the testing ability of ng-level mass [16–18]. The piezoelectric quartz sensor can be classified into two types—the mass effect type and the non-mass effect type [19–20]. The mass effect meets with the classical Sauerbrey formula [21]. The Formula (1) is as follows:

(1) $$\Delta F=-2.26\times 10^{6}\;\Delta {\mathrm{mf}}^{2}/A$$

where Δ*m* refers to the change of mass load (g); Δ*F* refers to the change of frequency arising from the change of Δ*m*; *f* refers to working frequency of quartz crystal (Hz); *A* refers to the area of electrode of quartz crystal (cm^2^); -refers to the decrease of frequency caused by the increase of mass. Nomura summarized the frequency formula of non mass effect type piezoelectric sensor contacting with liquid [22]. The Formula (2) is:

(2) $$\Delta f=-f_{0}^{3/2}{\left(\rho L\cdot \eta L/\pi \cdot \rho q\cdot \mu q\right)}^{1/2}$$

with *ρL* and *ηL* standing for density and viscosity of liquid, respectively, *ρq* referring to quartz density and *μq* standing for shear modulus.

In this research, we combined the mass effect and non mass effect type of piezoelectric quartz sensor in an independently developed piezoelectric agglutination sensor to detect the agglutination reaction of Fib. We established the judgment method for determining plasma agglutination reaction by piezoelectric agglutination sensor. Furthermore, the standard curve between plasma agglutination time and Fib concentration was established to determine Fib content quantitatively in the patients with coronary heart diseases.

## 2. Experimental Methods

**Figure 1.**
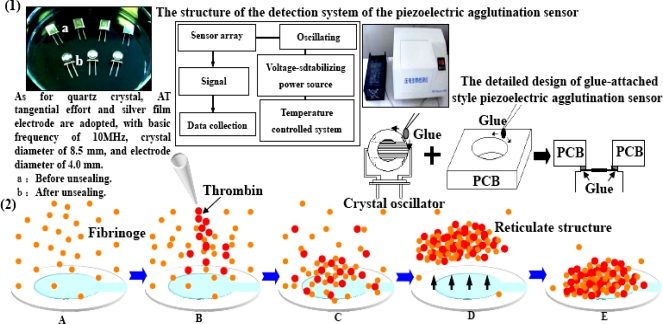
Components of piezoelectric quartz crystal oscillator and piezoelectric blood coagulation sensor detector and sketch map of determination of agglutination reaction of Fib. (1) The schematic structure of the equipment. (2) The schematic process of the measurement. A: Before the reaction, Fib is free in the detection pool on the piezoelectric quartz crystal oscillator surface, and the viscosity/density remains stable. B: Fib reaction reagent (thrombin) is added, and the reaction starts. C: After the agglutination reaction, fib gradually changes into fibrous protein monomer and complex, and the density and viscosity increase gradually and grid structure is formed; the mass load on crystal surface also increases; the common role of mass effect and non mass effect result in frequency changes. D and E: Near the agglutination reaction terminal, the blood clots shrink; the fibrous protein complex adhered to crystal surface leaves the surface; mass load decreases; the crystal frequency increases for a short period and then decreases with the deposit of blood clots, forming terminal peak.

Quartz crystal adopts AT tangential effort and silver film electrode, with a basic frequency of 10 MHz, crystal diameter of 8.5 mm, and electrode diameter of 4.0 mm (Chengdu Renhe Electronics Co., Ltd). The Quartz crystal oscillation frequency detector (independently developed) adopts an auto-stimulated oscillator circuit, TTL battery and a closed detection room of 12 cm × 7 cm × 4 cm. The detection room is equipped with facilities to resist external influencing factors such as moisture, temperature, electromagnetic radiation and gas shock. The detailed design of the detection room is shown in Figure 1. We designed a glue-attached style piezoelectric agglutination sensor. The printed circuit board) (PCB) (was used as the wall material, in which a hole was made and a circular fillister chiseled around the base of the hole. A crystal oscillator was mounted at the bottom of the hole before the fillister was gel-filled. Several quartz crystals and the detection rooms were deployed as a piezoelectric agglutination sensor array. Two electrodes were elicited and inserted in the corresponding positions of equipment. The used glue-attached style piezoelectric quartz sensor was pulled out and changed. A single quartz crystal was applied to determine the agglutination reaction for one time and could not be reused in a new reaction. Twelve signal paths were equipped with ACL-836 multi-functional tally card (Advantech Company). All 12 signal paths were being used. The rate of data acquisition for each signal path was once per second in this experiment, and could be set according to the needed experiment. The records of data acquisition for the 12 signal paths were not interrupted. The signal of each path was sent to a counter for counting. The tally card included 12 independent counters. The gate control was a 1 Hz square wave signal and had an independent frequency measurement unit. The start oscillation capacitance of the quartz crystal oscillators were 20 pf and the threshold voltage of the TTL battery unit was 0.7 V. PESA 2.0 analysis software (independently developed) can record and analyze frequency, time and change trend of frequency, *etc*. In addition, a STAGO fully-automatic agglutination analyzer (France STAGO Company) was also adopted. The accuracy of the STAGO equipment reached detection of 0.1 g/l Fib in plasma. Quartz crystal was rinsed three times with distilled water before usage. The main ingredient of the Fib reaction reagent (France STAGO Company) was thrombin (100 IU). The dilution buffer was 0.0284 M sodium barbital in 0.125 M sodium chloride (pH 7.35 ± 0.1). The basic reaction process was that blood plasma Fib turned into fibrin with excessive thrombin, and formed network structure by agglutination in the end.

One hundred and ninety-six coronary heart disease patients were grouped (the coronary heart disease group); 71, acute myocardial infarction; 64, unstable angina and 61, stable angina. The patients included 114 males and 82 females (average age is 55.7). They were hospitalized in the cardiovascular department of Southwest Hospital from 2006–2007. Ninety-eight healthy volunteers with negative coronary angiography were taken as the control, including 47 males and 51 females (average age is 48.5), coming from outpatients in the cardiovascular department in Southwest Hospital (2005–2007). 1.8 mL limosis vein blood was taken and added into the anticoagulant tube with 0.2 mL of 10^9^ mmol/L sodium citrate, and mixed well. The tube was centrifugated for 10 min with the velocity of 3,000 r/min, and the plasma in the top layer was collected for later use.

### 2.1. Constructing the platform of oscillation frequency agglutination detection by the piezoelectric agglutination sensor

The platform was mainly composed of quartz crystal oscillator, TTL oscillation frequency detection circuit, frequency signal collection and treatment system. The TTL frequency signal output interface of the piezoelectric quartz crystal frequency detector was connected with the frequency collection card interface of a built-in computer. The record and treatment of frequency data was automatically completed by PESA analysis software 2.0.

### 2.2. Constructing a standard fibrinogen detection curve by the piezoelectric agglutination sensor

The standard plasma was diluted according to the proportions of 1:10, 1:20, 1:30 and 1:40. Determinations were carried out in duplicate for all proportions, and the standard curve of concentration—time was established. The dilution proportion with the best determination result was chosen as the working concentration of the clinical specimen.

### 2.3. Determining Fib concentration in plasma from clinical specimens by the piezoelectric agglutination sensor

The subjects were divided into two groups—the coronary heart disease group and the control group. Samples of standard plasma with known Fib concentration and clinical plasma were diluted 1:20 with dilution buffer for detection. The plasma was placed into a 37 °C detection room to warm up. The quartz crystal was inserted into quartz crystal detection pool until the gas phase was stable. 60 μL of plasma was added into the quartz crystal detection pool, mixed evenly and left in the pool for 2–3 min. After the oscillation frequency of the crystal was stable, 30 μL of warmed Fib reaction reagent (proportion of 2:1) was added, rapidly mixed and the reaction started. The frequency was constantly detected until it was relatively stable. The gate time and interval for frequency collection was set to 1 s, and the frequencies and corresponding time were automatically collected and recorded. Fib concentration in plasma of clinical specimen by STAGO fully-automatic agglutination analyzer was determined according to the instructions of the instrument. Each determination was repeated three times and the mean value was taken.

### 2.4. Comparison between the method of the piezoelectric agglutination sensor and the paramagnetic particle method

The statistical software SPSS 11.5 was used for data treatment, the data were expressed as (*x̄* ± s); t examination was performed. The comparison of Fib concentration was carried out among the control group and the coronary heart disease group by one factor analysis of variance. LSD examination method was adopted for comparison between two groups.

**Figure 2.**
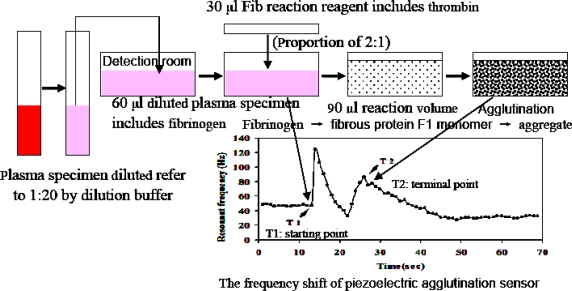
The basic process for measuring the agglutination reaction of Fib in plasma by piezoelectric agglutination sensor.

The basic process of measuring agglutination reaction of Fib in plasma by the piezoelectric agglutination sensor is shown in Figure 2.

## 3. Results and Discussion

### 3.1. Changes observed in trend of oscillation frequency of the quartz sensor during plasma agglutination

After the oscillation frequency of the crystal in quartz crystal detection pool was basically stable, Fib reaction reagent (thrombin) was added for the reaction to begin. The crystal oscillation frequency increased immediately, and then rapidly decreased in terraced shape with the fast increase of density and viscosity of the reaction system. After this oscillation frequency decrease near the terminal of the plasma agglutination reaction (*i.e.*, the terminal peak), it then was gradually stable. Fib reaction reagent (thrombin) was added to start agglutination reaction. Due to even shock, increase of density and viscosity and rapid increase of frequency, the starting point of the frequency increase was the starting point of the agglutination reaction (starting point peak). As the agglutination reaction proceeded, Fib changed into fibrous protein F1 monomer and complex, and the density and viscosity of the reaction system increased constantly. Due to the adhesion of fibrous protein F1 monomer and complex to crystal surface, the mass load increased. Under the common roles of mass effect and non mass effect, the crystal frequency decreases in terraced shape. Near the agglutination reaction terminal, the density and viscosity of reaction system stop increasing. At the same time, due to shrinkage of blood clots, the fibrous protein complex adhered to the crystal surface then leave the surface, mass load decreases, and the crystal frequency increases. After a short period, with deposit of blood clots, the crystal frequency decreases, and the turning point of frequency is the terminal of agglutination reaction (terminal peak). The rule of the quartz crystal oscillation frequency changes reflects the whole process of the agglutination reaction objectively, is coincident with the mass effect and the non-mass effect of quartz crystal, and provides objective basis for correct judgments of starting point and terminal of agglutination reaction.

### 3.2. Establishing detection method of Fib (terminal judgment method)

**Figure 3.**
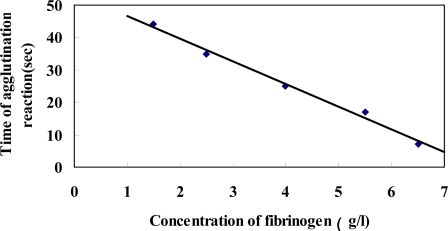
Standard curve of Fib detected by the piezoelectric agglutination sensor.

The change trend of quartz sensor oscillation frequency during the agglutination course shows that the addition of the reaction reagent to start the reaction is the starting point of agglutination reaction (T1), as shown in Figure 3. As the agglutination reaction proceeds, the oscillation frequency increases and then decreases. The turning point (terminal peak) is the terminal of agglutination reaction (T2). The corresponding time of starting point and terminal of agglutination reaction are collected and recorded. The period between the starting point and the terminal point (T2-T1), the agglutination period, is calculated. If the reaction conditions, such as pre-temperature treatment, equipment preparation, the sample treatment, fast mixture and so on, are properly controlled, the terminal peak (T2) is sure to be obtained. At the same time, we use the curve fitting model and maximal-curvature judgment to help judge the reaction termination. Because of the hand operation, samples must be mixed quickly (<2 s) after Fib reaction reagent is added, otherwise the judgment of the reaction terminal point will be influenced. The agglutination reaction starts immediately after Fib reaction reagent (thrombin) is added. Therefore, mixing as fast as possible can effectively reduce the possible deviation of the judgment of reaction terminal peak (T2) evoked by long time mixing. The study was designed to detect the starting point (T1) and the terminal point (T2) with an interval and the inter-medial complicate agglutination process ignored, so the 2 s manual mixing can ensure the intensive mixing of the 90 μL reaction system.

In the liquid phase environment, the piezoelectric quartz sensor is highly sensitive to the mass load on the crystal surface and to the property changes of the liquid phase reaction system such as density, viscosity and electrical conductivity [23,24]. Combining the mass effect and non mass effect of piezoelectric quartz sensor, we can perform dynamic real-time monitoring of the agglutination reaction and determine the terminal point of agglutination reaction according to the rule of oscillation frequency changes.

### 3.3. Standard curve of Fib detected by the piezoelectric blood coagulation sensor

Ideally, the agglutination reaction time of diluted standard Fib corresponds with the concentration of corresponding standard sample. As a result, the standard concentration – time curve is established. The frequency collection analysis software records the frequency automatically at the set time point. The agglutination time is calculated according to the time between the starting point and terminal point and the concentrations of the corresponding standard Fib are known. Standard Fib concentration is taken as the X-axis, and agglutination time as Y-axis (Figure 3). The standard curve of Fib concentration—agglutination reaction time is established by linear regression. The agglutination reaction time of unknown samples can be measured by the piezoelectric agglutination sensor, and corresponding Fib concentration can be obtained by the curve of Fib concentration—agglutination reaction time. In the process of constructing the standard curve of Fib concentration—agglutination reaction time, we found that the linearity is best when the plasma is diluted to 1:20, therefore we chose the dilution ratio (1:20) to detect clinical samples.

### 3.4. Clinical sample detection

**Figure 4.**
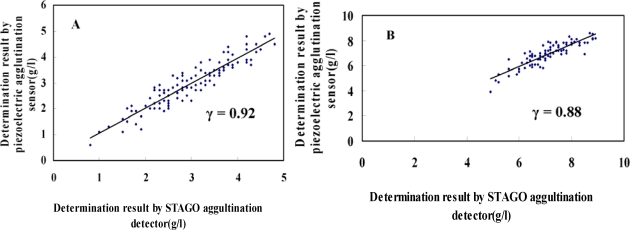

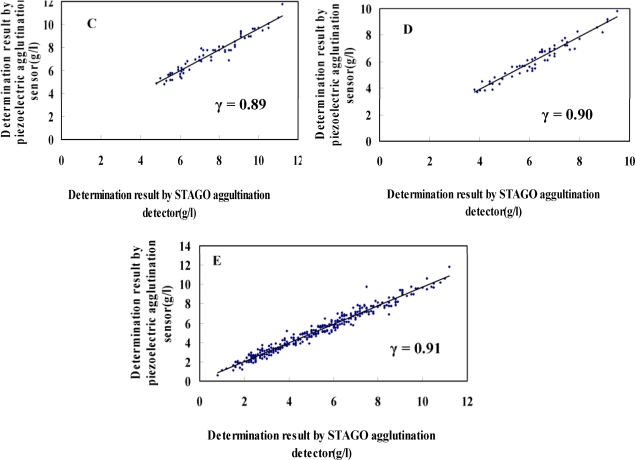
Comparing Fib results by piezoelectric agglutination sensor and STAGO agglutination detector methods. A: 98 healthy individuals as the control group. B: 71 patients, acute myocardial infarction. C: 64, unstable angina. D: 61, stable angina. E: 294 samples, including 196 samples from patients with coronary heart diseases and 98 healthy individuals in the control group.

Fib concentration in plasma was detected for 196 samples from coronary heart disease patients and 98 healthy people in the control group by piezoelectric quartz sensor. At the same time, STAGO agglutination analyzer was adopted for parallel detection and linear analysis, as shown in Figure 4. The data were divided into four groups including acute myocardial infarction, unstable angina, stable angina and healthy. Correlation analysis was performed in each group and different coefficient correlations were obtained (0.92, 0.88, 0.89 and 0.90, respectively). The total coefficient correlation was 0.91. The result of Fib by the piezoelectric quartz sensor method coincided with that by STAGO agglutination analyzer. The method of piezoelectric agglutination sensor is a novel method of detecting Fib in plasma. The comparison of some operational parameters of the two methods is shown in Table 1.

**Figure 5.**
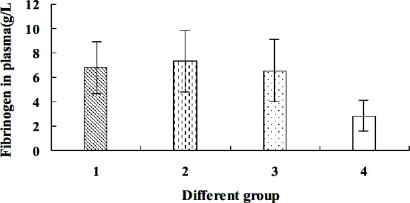
Fib concentration in plasma of patients in different groups: 1. Acute myocardial infarction group; 2. Unstable angina group; 3. Stable angina group; 4. Healthy control group.

Fib concentrations in plasma: patients with acute myocardial infarction: (6.8 ± 2.5) g/L; patients with unstable angina: (7.3 ± 2.7) g/L; patients with stable angina: (6.7 ± 2.8) g/L; healthy control group: (2.9 ± 1.2) g/L. The Fib concentrations from patients with acute myocardial infarction, unstable angina and stable angina through one factor analysis of variance are higher than those of the control group (P < 0.05); the comparisons among groups of acute myocardial infarction, unstable angina and stable angina, and the differences do not bear significant statistical meanings (P > 0.05), as shown in Figure 5. The results indicate that plasma Fib is related to coronary heart diseases and is an independent dangerous factor inducing coronary heart diseases. The mechanism of coronary heart disease induced by high plasma Fib are as follows [25,26]: (1) Fib changes into fibrous protein, which promotes platelet aggregation and deposition on the vessel walls, as well as the incurrence and development of coronary atherosclerotic score; (2) the bridging role of giant molecule Fib and fibrous protein increases the viscosity of blood and the probability of thrombus; (3) Fib prohibits the formation of nitric oxide synthase and nitric oxide of smooth muscle cells, thus promotes coronary vasospasm and causes damage to blood vessels.

## 4. Conclusions

In this research, we determine Fib concentrations through detecting the plasma agglutination time from patients with coronary heart diseases by use of a piezoelectric agglutination sensor. We establish the terminal judgment method of monitoring plasma agglutination reaction through piezoelectric agglutination sensor, as well as the dose-effect relationship between plasma agglutination time and Fib for quantitative determination of Fib content. The result is consistent with the determination results of the currently adopted STAGO blood coagulation analyzer. The related coefficient of the two methods is 0.91. Therefore, the method of the piezoelectric agglutination sensor is another new method to determine Fib. The result by piezoelectric agglutination sensor method is in good concordance with that by STAGO paramagnetic particle method. The method is reliable and can be used in clinic. The method of piezoelectric agglutination sensor has some merits such as operation convenience, portability due to small size, low cost and quick detection. The Fib result in the group of coronary heart diseases is significantly higher than that of the healthy control group (P < 0.05). The results suggest that high Fib is closely related to incurrence, development and prognosis of coronary heart diseases.

## Figures and Tables

**Table 1.** Comparison of some operational parameters of the Piezoelectric Agglutination Sensor and the STAGO paramagnetic particle methods.

	**Analysis time**	**Analysis precision**	**Required sample volume**	**Cost of test**
**Piezoelectric Agglutination Sensor**	**<5 min**	**0.1 g/L**	**3 μL**	**1 $**
**STAGO paramagnetic particle method**	**10 min**	**0.1 g/L**	**10 μL**	**5 $**
